# Fitness related effects of titanium dioxide nanoparticles and glyphosate exposure on *Cardiocondyla obscurior*

**DOI:** 10.1007/s11356-025-37388-y

**Published:** 2026-01-23

**Authors:** Danae Nyckees, Raquel Gonzalez de Vega, Reinhard Sittinger, David Clases, Dalial Freitak

**Affiliations:** 1 Institute of Biology, Karl-Franzens-Universitat Graz, Graz, Austria; 2 Institute of Chemistry, Karl-Franzens-Universitat Graz, Graz, Austria

**Keywords:** Titanium dioxide nanoparticles, Herbicide, Combined stressor, *Cardicondyla obscurior*, Ant fitness, LA-ICP-TOFMS

## Abstract

Insects are essential for ecosystem functioning and their rapid decline is alarming. While it is evident that various pollutants such as pesticides play a crucial role in the disappearance of insects, knowledge on the effects of specific inorganic compounds such as nanoparticles (NPs) are less investigated and little is known about their direct and indirect impacts. Titanium dioxide nanoparticles (TiO_2_NPs) are abundantly found in the environment but their effects on insects is little known, as well as their role as vectors for other chemical stressors. In this study, we investigated lethal and sublethal effects of TiO_2_NPs on *Cardiocondyla obscurior* ants alone or combined with the herbicide glyphosate. We measured survival, brood production, gut endosymbiont densities, reactive oxygen species (ROS) accumulation, morphological features and elemental TiO_2_ distribution in ants chronically exposed to environmentally realistic concentrations of TiO_2_NPs and/or glyphosate. We found that proportional brood distribution was altered by the presence of the stressors and it was time dependent. Moreover, we observed a synergistic effect between TiO_2_NPs and glyphosate with bigger newly produced queens, altered gut endosymbionts densities and accumulation of TiO_2_NPs. These results shed light on the ecotoxicological effects of TiO_2_NPs ingestion and highlight the importance of understanding the synergistic effects of pollutants.

**Supplementary Information:**

The online version contains supplementary material available at 10.1007/s11356-025-37388-y.

## Introduction

Insects play crucial roles in maintaining stable and healthy ecosystems by providing numerous essential services that benefit both the environment and human society (Losey and Vaughan 2006). Yet, insect populations are experiencing severe declines as shown in multiple studies (e.g., Grubisic et al. 2018; Wagner 2020; Sanchez-Bayo and Wyckhuys 2021). This decline has raised global concern about the risks it engenders (Goulson 2019; Van Der Sluijst, 2020). The cause of this decline, often referred as "Insect Apocalypse" or "Ecological Armageddon", is multiple and complex but among the key factors are environmental pollutants (Wagner 2020), including pesticides (van Lexmond et al. 2015) and heavy metals (Singh et al. 2022).

Certain heavy metals are found in the form of nanoparticles (NPs). They exist naturally (Abdallah et al. 2020; Arunmetha et al. 2019) but are also increasingly manufactured – intentionally and unintentionally (Gonzalez-Pech et al. 2019)– and constitute a class of contaminants which is little considered (Williams 2022). One example for naturally occurring but also anthropogenically manufactured heavy metal particles are titanium dioxide nanoparticles (TiO_2_NPs). They are known for their unique physical and chemical properties, such as high refractive index, strong UV light absorption, and photocatalytic capabilities (Akakuru et al. 2020). The production and application of TiO_2_NPs have been exponentially increasing, being integral to products ranging from paints to pharmaceuticals, consumer products and even extending to food industries (Thakur et al. 2024). TiO_2_NPs were detected in multiple aquatic environments with concentrations ranging from 0.01 to 13.6ppm (e.g.: Brunelli et al. 2013; Bitragunta et al. 2017 et al., 2024; Fernández-García et al. 2025). However, the environmental fate of TiO_2_NPs and their interaction with biotic systems remains understudied and calls for more research due to rising concerns about their potential ecotoxicological effects (Du et al. 2019).

There have been few studies that investigated the toxicological effect of TiO_2_NPs, providing evidence of detrimental consequences on terrestrial and aquatic animals (Federici et al. 2007; Dabrunz et al. 2011; Libralato et al. 2013; Wang et al. 2013, 2019). TiO₂NPs interact with organisms through various mechanistic pathways, impacting cellular and physiological functions. TiO₂NPs can enter cells via multiple endocytic pathways (Thurn et al. 2017) and generate reactive oxygen species (ROS; Chan et al. 2011), interfere with mitochondrial function (Ikram et al. 2021) and disrupt the cytoskeleton (Chan et al. 2011). Additionally, TiO₂NPs have been shown to modulate gene expression and signalling pathways (Ikram et al. 2021) and to pass through the intestinal barrier and accumulate in various organ (Koeneman et al. 2010). In insects, the mode of action of NPs is not well studied (Benelli 2018) but exposure to TiO_2_NPs has been shown to induce damage in brain structures of honeybees (Shahzad and Manzoor 2024), as well as oxidative stress and DNA damage (Sabat et al. 2016; Demir 2020; ha et al. 2023). Among other effects, exposure to TiO₂NPs lead to deficits of locomotor behaviour in *Drosophila, *altered the composition and structure of gut microbes (Li et al. 2020; Papa et al. 2021) and affected brood developmental time (Posgai et al. 2011; Jovanovic et al., 2016; López-Muñoz et al. 2019). Moreover, the synergistic effects of NPs with other pollutants are often overlooked. Synergetic effect of TiO_2_NPs with deltamethrin, a pyrethroid pesticide, was recently observed on honeybees (Shahzad and Manzoor 2024). This raises the concern about the synergistic effects between TiO_2_NPs and pesticides in general, another widespread class of contaminants.

Most commonly used pesticides include glyphosate-based herbicides. They are recognized as the most widely used herbicides globally, frequently making headlines due to the controversies surrounding their environmental and health ramifications (Kudsk et al., 2020). With a host of studies appraising glyphosate´s safety profile, its persistence in ecosystems along with its extensive application in agriculture and urban areas has been shown to result in inadvertent exposures among non-target organisms, including insects (Thompson et al. 2022). Glyphosate is not directly toxic to insects as the molecule targets an enzyme that insects lack (Cole 1985). Nonetheless, exposure to glyphosate has been shown to be detrimental to insects (e.g., Milan et al. 2018; Battisti et al. 2021; Smith et al. 2021). Glyphosate is still in use in the European Union and will remain until at least 2033 as the regulatory authorities determined in 2023 that there is no scientific or legal justification for a ban (European Commission 2025). However, member countries are permitted to apply different rules, and some nations have introduced partial bans (Phytoweb 2025).

In light of the recent constitutional decision, the continued large-scale use of glyphosate necessitates a deeper understanding of its effects—particularly its interactions with other increasingly prevalent environmental contaminants, such as nanoparticles. However, TiO_2_NPs and glyphosate are not inherently found together as they are used for different purposes and their co-occurrence in the environment has not been targeted in studies so far. Nevertheless, the co-occurrence is predicted to happen as on the one hand, TiO_2_NPs have a high abundance naturally and on the other hand, manufactured NPs end up together with glyphosate in wastewater and soil (Sun et al. 2016; Feng et al. 2020). The inclusion of TiO_2_NPs in commercial products is contributing to their build-up in sewage sludge which is then utilized as fertilizer on agricultural lands in numerous countries (Gottschalk et al. 2013; Sun et al. 2016; Sharma et al. 2017). Furthermore, nanotechnology is more and more incorporated into agricultural techniques. TiO_2_NPs are now proposed as plant growth promoter (Mattiello and Marchiol 2017; Martin et al. 2021), to stimulate seed germination (Khamova et al. 2021), as fertilisers, pesticides, remediation and biosensors (Rodriguez-Gonzalez et al. 2019; Thiagarajan et al., 2021). This practice would make co-exposure to pesticides and TiO_2_NPs unavoidable and there is few information regarding the interactions of these two chemicals nor their by-products. There is, nevertheless, a couple of studies investigating the photodegradation of glyphosate by TiO_2_NPs (Gupta and Tripathi 2011; Marconi et al. 2025) demonstrating that these chemicals are affecting each other.

Insects are likely to encounter both TiO_2_NPs and glyphosate in nature through deliberate field applications or unintentionally, as the environmental abundances continue to rise. They can be exposed through various pathways, including ingestion through nectar and pollen (Thompson et al. 2022) and topical contact (Abraham et al. 2018). Ants (Hymenoptera: Formicidae) are good model organism for ecotoxicological investigations. They have been shown to be suitable as bioindicators (King et al. 1998; Andersen and Majer 2004; Majer et al. 2007) and are sensitive to environmental changes induced by pesticide and heavy metal pollutants (Ribas et al. 2012; de Barros et al. 2022). They are vital for the maintenance of terrestrial ecosystems as they contribute to a multitude of ecological services such as predation, soil aeration, decomposition, and seed dispersal (Crist 2009; Wills and Landis 2018). Ants are an abundant and widespread group of insects, and many species are soil-nesting.

Recognizing the pressing need to investigate the impacts of contemporary pollutants on organisms, this study seeks to elucidate the cumulative and synergistic effects of TiO_2_NPs in tandem with glyphosate on *Cardiocondyla obscurior. C. obscurior* is well-documented regarding its biology, relevance in environmental monitoring, and its susceptibility to environmental pollutants (Heinze et al. 2006; Leponiemi et al. 2022) making it a great model species for the purpose of this study. We hypothesize that the interaction between TiO_2_NPs and glyphosate may amplify the toxicity beyond their individual effects. It is paramount to explore the nature of such interactions within a controlled setting to predict and mitigate potential risks in natural habitats, as organisms will be exposed to several compounds simultaneously.

This research evaluates multiple parameters related to colony fitness after oral short-term chronic exposure to TiO_2_NPs and glyphosate mimicking environmentally relevant concentrations. Mortality rates were assessed to examine a direct measure of toxicity. Brood production and morphometric measurements of newly emerged queen were assessed to gain insight into the sublethal effects impacting reproductive success. Moreover, biochemical assays quantifying free radicals were performed to reveal stress attributed to ROS — a signature response to pollutant-induced stressors (Ahmad 1995; Lavarias et al., 2017). The examination of endosymbiont densities was also assessed as symbiotic relationships are essential for insect physiology (Engel and Moran 2013) and sensitive to environmental pollution (Motta et al. 2018; Blot et al. 2019; Abrar et al. 2022). Finally, the measurement of TiO_2_ levels and their distribution within the ant's body was conducted to assess the extent of bioaccumulation.

## Material and methods

### Study species

Colonies of *Cardiocondyla obscurior* (subfamily of Myrmicinae) originally collected in Okinawa, Japan in 2010 and reared in the laboratory since then were used for this experiment. *C. obscurior* is native to Southeast Asia and has established itself throughout the tropics and subtropics via human activities (Schifani et al. 2024). They typically form small colonies and forages solitary (Heinze et al. 2006). The developmental process of *C. obscurior* includes several stages starting with egg, followed by three larval instars, the pupal and, lastly, the adult stage (Schrempf and Heinze 2006). Their lifespan is influenced by multiple factors (Heinze et al. 2006) but in the lab queens live for about 24 weeks, workers 8, winged males 2 and ergatoid males live for approximately 6 weeks (Fuessl et al. 2015).

### Experimental set-up 1 and feeding treatment

Twelve independent stock colonies were divided into six fragments each (*n* = 72) and transferred to experimental nests. Each fragment consisted of 40 workers and two queens. Ants were picked randomly, resulting in a mixed age cohort. The experimental nest was made of a 9 cm petri dish with the bottom half-plastered and a ventilation hole in the lid. Experimental nests were kept under controlled conditions (LD 12:12 h, 25 °C and 70% relative humidity). Ants that died during the transfer to the experimental nest were replaced a week later, this replacement started the observation period. The number of replaced ants was not significantly different between the treatments, hence it is expected that the effect of replacement is homogenic. The model and test are exposed in the supplementary Table 1.

Experimental colonies were fed with a 3:1 mixture of honey solution (750mg honey diluted in 250 µl of filter H_2_O) and glyphosate (Sigma-Aldrich, Missouri, United States) dissolved in distilled water (dH_2_0) and/or dioxide titanium nanoparticles (21nm primary particle size, 80%/20% anatase/rutile) from Sigma-Aldrich, (Missouri, United States). In total six feeding treatments were set up: 1) Control (CO) – no supplement in the dH_2_0; 2) 5 µg/ml glyphosate (GL); 3) 1 µg/ml TiO_2_ (LT); 4) 500 µg/ml TiO_2_ (HT); 5) 5 µg/ml glyphosate and 1 µg/ml TiO_2_ (LTGL); 6) 5 µg/ml glyphosate and 500 µg/ml TiO_2_ (HTGL). For the combined treatments, the chemicals were directly mixed together within the honey solution. Glyphosate and TiO_2_ chosen concentrations were aimed to reflect realistic environmental concentrations (Giesy et al. 2000; Herbert et al. 2014; Thompson et al. 2014; Sun et al. 2016).

**Fig. 1:**
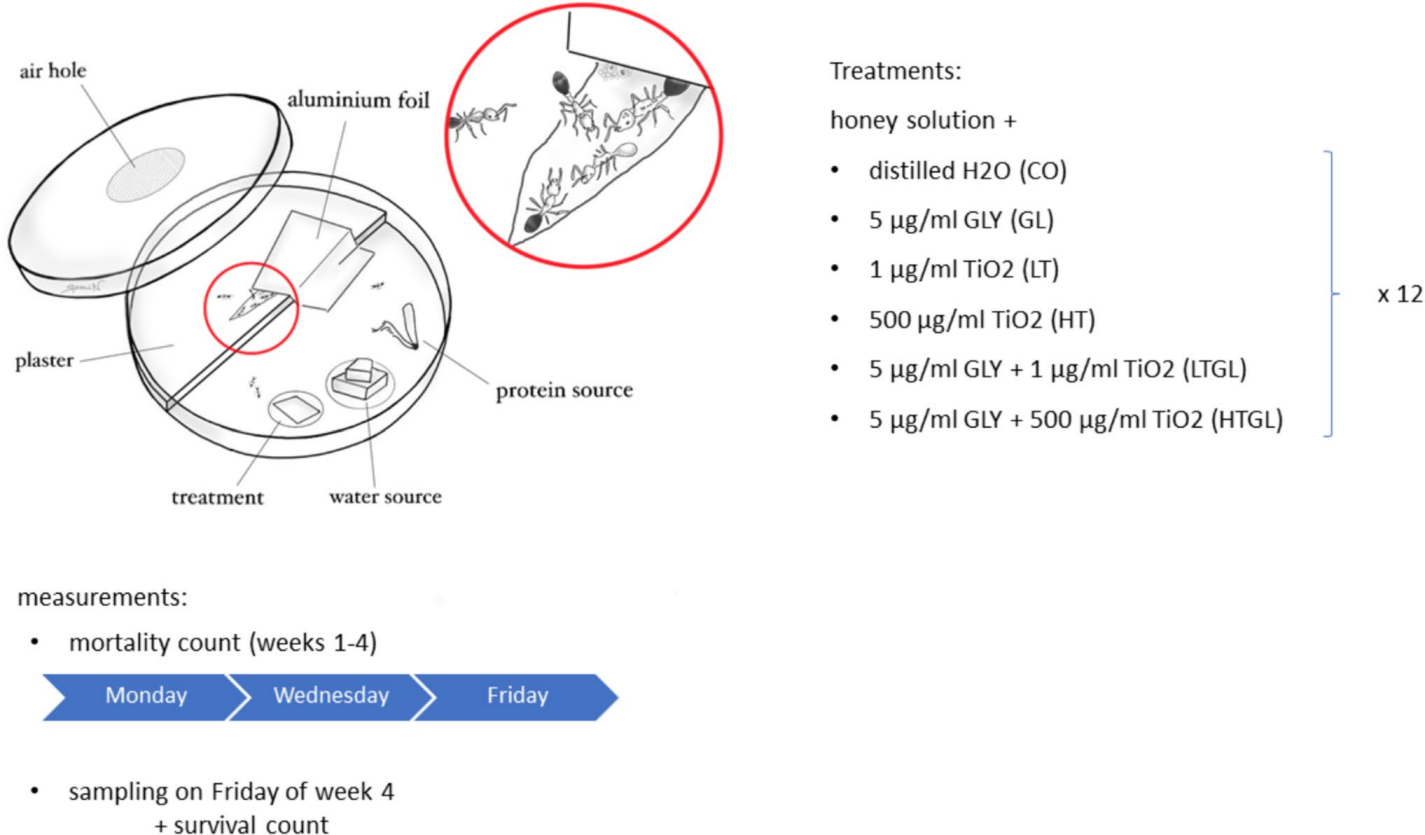
Schematic experimental design. Drawing by Romi Nerot

Fresh food was provided three times a week by giving 25 µl of the feeding treatment pipetted on a small square of paper towel (1 × 1 mm) and a protein source (one cockroach leg twice a week and five fruit flies once a week). Cockroaches were purchased online (https://www.terraristikshop.net/) and fruit flies were reared in the laboratory following published protocols (Bass et al. 2007; Grandison et al. 2009). A fresh food stock was prepared every week and kept at 4°C. The food was heated to 40 °C and vortexed prior to feeding. Distilled water was provided by wetting a Wettex-strip every day of feeding. The experimental design is shown in Fig. 1.

### Mortality and brood count

The effect of the treatments on survival was measured by counting mortality three times a week during the feeding for four weeks. Dead individuals were removed. Queen survival per experimental colony was counted at the end of the four weeks. The effect of the treatments on brood production was measured by counting eggs, larvae and pupae each week. Pupae were removed from the experimental nests to keep the adult number constant.

### Endosymbiont density

The effect of the treatments on the worker gut endosymbiont was assessed by measuring the density of two obligate bacteria *Wolbachia* and *Cand*. *Westeberhardia*. After four weeks, five workers from each experimental nest were collected and placed into sterile 1.5ml Eppendorf tubes and stored at −20°C.

We targeted the cytochrome oxidase gene (Ün et al. 2021) for *Wolbachia* and the nrdb gene (Klein et al. 2016) for *Westeberhardia*. Elongation factor 1-alpha 1 gene was used as reference gene (Klein et al. 2016). Primers sequences are shown in supplementary Table 2.

The relative abundance of symbionts was determined by first extracting genomic DNA and then measuring it with quantitative polymerase chain reaction. DNA was isolated from the samples using the QIAamp DNA Micro Kit (Qiagen, Venlo, The Netherlands) and following the manufacturer protocol. The samples were lysed overnight at 56 °C with the buffer ATL and proteinase K. Before purification, 1µl of RNA carrier was added to increase DNA yield. 45µl of the buffer AE was added 5 min before the final centrifugation. DNA quantity was determined with a Nanodrop One^c^ (Thermo Fisher Scientific, Massachusetts, United States).

Quantitative real-time PCR was performed in 384-well hardshell PCR plates in a CFX384 Real-Time System (Biorad, California, United States) using the PerfeCTa® SYBR® Green FastMix® ROX (Quantabio, Massachusetts, United States) and a total sample volume of 20μl, consisting of 14μl master mix (10μl SYBR plus 4μl primer set) and 6μl of the sample with 3μg/μl of DNA (95°C 3 min; 40 × 95 °C 5s, 60 °C 20s; 95 °C 10s). Each sample was analysed in two technical replicates.

### Reactive oxygen species quantification

The effect of the treatments on the production of ROS was assessed. After four weeks, three workers from each experimental nest were collected and placed into sterile 2 ml Eppendorf tubes and stored at −20°C in 300µl of 2mg/ml PBS 3-amino-1,2,4-triazole solution (A8056, Sigma-Aldrich, Missouri, United States) until analysis.

Samples were homogenized with the TissueLyser II 168 (Qiagen, Venlo, The Netherlands) for 3 min at 30Hz. The samples were then centrifuged at 14000rpm for 3 min at 4 °C and the supernatant was collected for analysis.

The ROS content was measured using the Amplex Red Hydrogen Peroxide/Peroxidase Assay Kit (Invitrogen, Massachusetts, United States). The analysis was done according to the manufacturer’s protocol. The fluorescence of the samples was read on ƛ_ex_540nm – ƛ_ex_ 590nm after excitation in a microplate reader (SpectraMax iD3, Molecular Device, California, United States).

The protein content was also measured to consider for the size variation between sampled ants and the standardisation of the ROS samples. The Bicinchoninic Acid Kit (Invitrogen, Massachusetts, United States) was used following the manufacturer’s protocol. The absorbance of the samples was read at 562nm in a microplate reader (SpectraMax iD3, Molecular Device, California, United States).

### LA-ICP-TOFMS to resolve Ti distributions in ants

Localization and quantification of TiO_2_ in single ants was assessed after four weeks of feeding treatments by laser ablation-inductively coupled plasma-time of flight mass spectrometry (LA-ICP-TOFMS). Six ants from each treatment were fixed on a microscope slide using double sided tape. The ants were glued on their ventral side and arranged carefully to be flatly attached without being squished.

LA-ICP-TOFMS experiments were performed with an Analyte G2 excimer (193nm) laser ablation system (Teledyne CETAC, Omaha, NE, USA) equipped with an aerosol rapid introduction system (ARIS, Teledyne CETAC) coupled to a Vitesse ICP-TOFMS platform by Nu Instruments (Wrexham, UK). The plasma was operated at 1.35 kW, helium (12ml min^−1^) and hydrogen (10ml min^−1^) were used as cell gases and the typical flow rate for nebulizer gas was 1.25L min ^−1^. Data acquisition was performed using the Nu Codaq software (Nu Instruments). The monitored mass range was 20-234amu while selecting a blanking region of m/z 32.5–43 to avoid high intensity elements overloading the detector.

The LA-ICP-TOFMS set-up was tuned for maximum sensitivity before each measurement using the reference material NIST 612 “Trace Elements in Glass”. The laser beam spot size was adjusted to 10µm, with a frequency of 100 Hz, a dosage of 4 generating and a fluence of 2 J cm^−2^. Helium was used as carrier gas (99.999% purity, Messer Austria GmbH) with a flow rate of 0.5L min-1 (0.25L min^−1^ cell gas, 0.25L min^−1^ ablation cup gas).

Gelatine for LA-ICP-TOFMS standard preparation was obtained from MM Ingredients (Wimborne, Dorset, UK) and background levels of cations were reduced in an extraction step employing an ion exchange resin (Amberlyst® 15 hydrogen form, Sigma Aldrich). Element standards (1000µg/ml, Single-Element ICP-Standard-Solution Roti®Star) were diluted to working concentrations using ultra-pure water (18.2MΩ cm, Merck Millipore, Bedford, USA) and spiked into gelatine to yield external calibration standards. External calibration was carried out by manufacturing gelatine standards following a protocol previously described by Westerhausen et al. (10.1039/C9AN01580A). Calibration curves for Ti were constructed by plotting the signal intensity obtained from LA-ICP-TOFMS against the concentrations of the standards. The exact elemental levels of the gelatine standards were determined by solution nebulization ICP-MS/MS after gelatine digestion.

LA-ICP-TOFMS data was visualised using the Pew^2^ software (10.1021/acs.analchem.1c02138). Background signals and tissue artifacts in the images were segmented and removed using a K-means clustering algorithm (k = 3, t = 1). This approach also facilitated the accurate identification of regions of interest, enabling precise localization of titanium (Ti) accumulation.

**Fig. 2:**
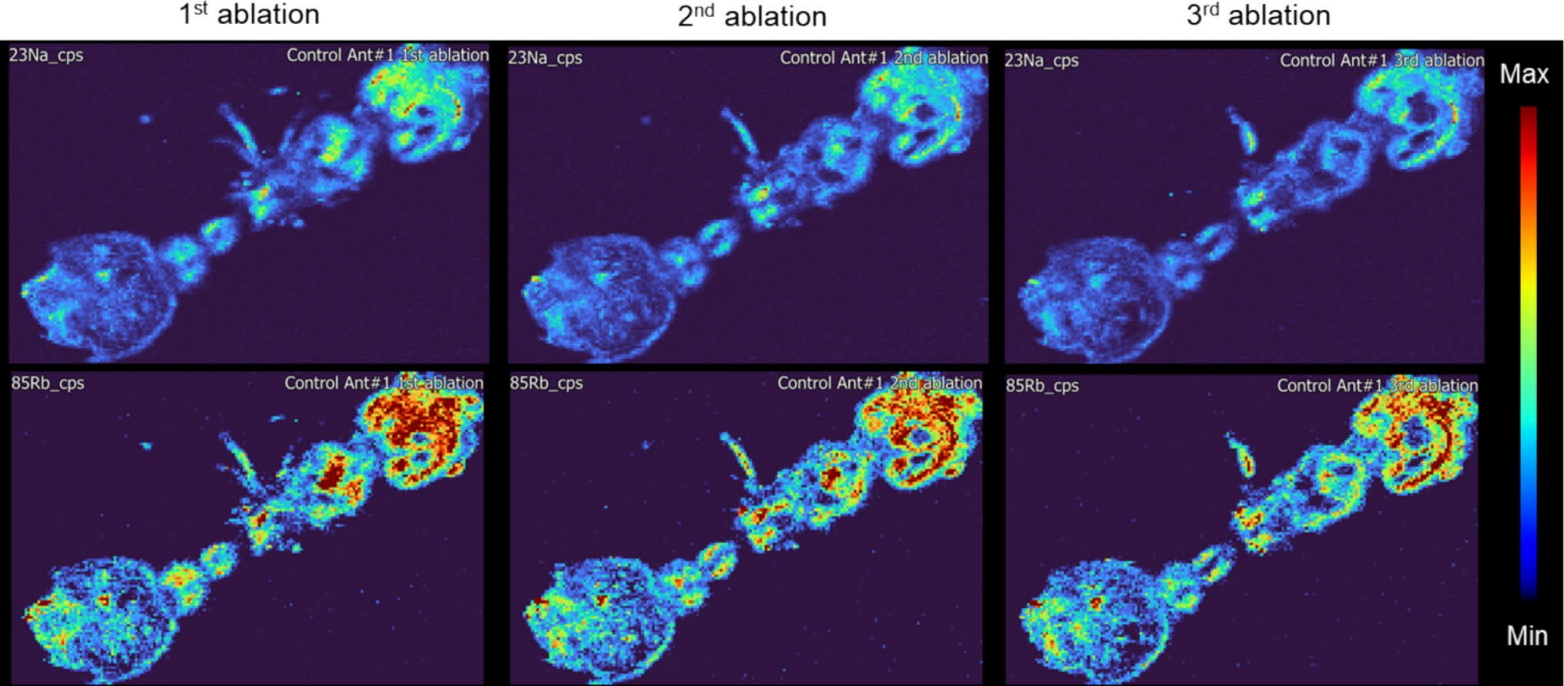
Repeated ablation of a single ant showing reproducible distributions. Na and Rb were chosen as representative elements as they are distributed across the entire body

It was first necessary to remove the exoskeleton. This step was relevant because the exoskeleton, composed of chitin and other organic compounds, may act as a physical barrier, which on one side may accumulate TiO_2_NP during exposure experiments and on the other side, prevent the laser ablation process from effectively reaching and analysing internal tissues. This was achieved through a pre-ablation step, providing direct access to the internal tissues. Here, the laser ablation process removed the upper layers of the ant. Na and Rb showed a relatively homogenous distribution through the ant tissues and their qualitative distribution were selected as indicator for the ant’s location and dimensions. Three ablation passes were carried out to study the possibility to remove the outer layer and to generate repeatable elemental maps as shown in Fig. 2. In subsequent analysis, the first elemental map was discarded and only the second was saved for further evaluating the presence and accumulation of Ti.

**Fig. 3:**
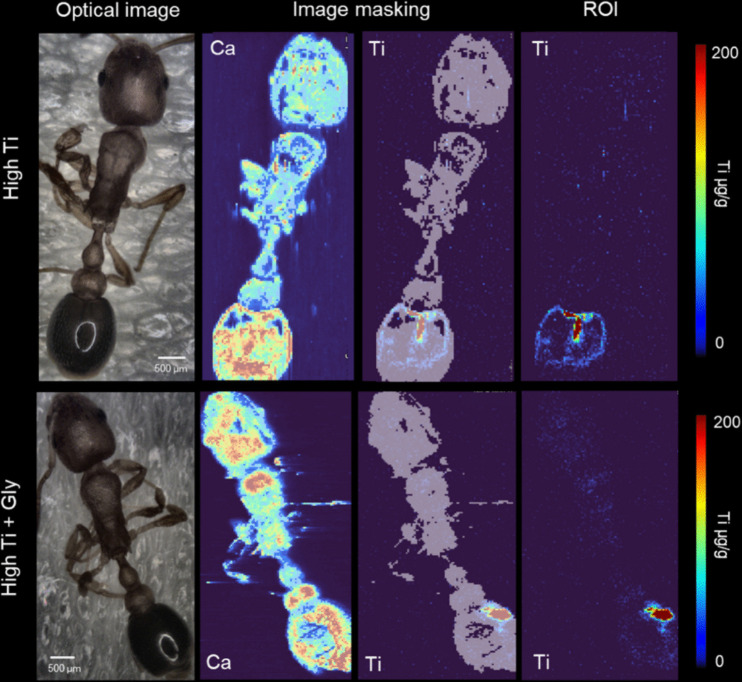
Titanium distribution of two representative ants fed with high TiO_2_NPs (top) and, high TiO_2_NPs and glyphosate bottom) for four weeks. From left to right, the pictures show the optical image of the intact ant, a masking using Ca to determine the ant structure and ROI of the Ti accumulation within the abdomen

Titanium distribution in two representative ants exposed for four weeks to high concentrations of TiO₂ nanoparticles and a combination of high TiO₂ nanoparticles and glyphosate are illustrated in Fig. 3. From left to right, the images show the optical image of the intact ant, a calcium-based mask used to define the ant structure and determine the region of interest (ROI), and the corresponding Ti distribution within the abdomen.

### Experimental set-up 2 and morphometrics

A separate experiment was conducted to measure the effect of TiO_2_ and GL on the body size of the newly produced queen from exposed mother queens. For this, fifteen independent stock colonies were divided into four fragments each (*n* = 60) housed in separate nests. Each fragment consisted of 50 workers and 5 queens. Ants that died after transfer to their experimental nest were considered to die due to the handling stress and were replaced the next day. This replacement started the feeding with spiked food. Male pupae were removed on a weekly basis to avoid mating with newly emerged queens. Male pupae were distinguished based on the features described by Schrader et al. (2015). Experimental colonies were fed for 5 months, following the feeding protocol previously described, at the end of which, virgin queens were collected and kept at −20°C until further analysis. In total, there was four feeding treatments: 1) Control (CO) – no supplement in the dH_2_O; 2) 5 μg/ml glyphosate (GL); 3) 1 μg/ml TiO_2_ (T); 4) 5 μg/ml glyphosate and 1 μg/ml TiO_2_ (TGL).

For the morphometric measurement, virgin queens were glued to paper cards. The widths of the head, thorax and petiole were measured as well as the length of the head and thorax. The head was measured above the eyes on an anterior view. Petiole was measured dorsally at the widest part. Thorax was measured dorsally at the widest part of the mesoscutum anterior to the wings. Ants were imaged using a stereomicroscope (Nikon SMZ 25, Tokyo, Japan) and images were acquired using the software NIS-Elements BR 5.20.00 (Nikon, Tokyo, Japan). Image processing and morphometric measurements were performed in Fiji (v. 1.52).

### Statistical analysis

All statistical analysis were performed with the R software (version 4.3.1, R Core Team 2023). Figures were drawn using *ggplot2* (Wickham 2016). For all linear models, the assumptions for linearity, homoscedasticity and normality of residuals were inspected visually and with DHARMa-package (Hartig 2022). The effect of the fixed factors was determined with an ANOVA from the car package (Fox and Weisberg 2018). Pairwise comparisons were conducted using the *emmeans* package (Lenth 2022) with a Tukey’s p-value adjustment for multiple comparisons. The experimental colony 16 (treatment: GL) was omitted from statistical analysis due to the high number of escaping ants.

The survival data were analysed using cox proportional hazard models from the *coxme* package (Therneau 2022). We used the number of individual ants which died on each counting day as the response variable, with feeding treatment as a fixed factor. Colony of origin and colony number were used as random factors (Survival model: (Day, Survival) ~ treatment + (1|original colony) + (1|colony number)).

The queen number was analysed using the non-parametric test of Kruskal–Wallis as normality was not met. We used the number of queens remaining as the response variable, with feeding treatment as a fixed factor.

The brood data were analysed using general linear mixed models from the *glmmTMB* package (Brooks et al. 2017). The weekly brood production was assessed using a negative binomial distribution where we used the brood count as the response variable, with week, feeding treatment and brood stage (egg, larvae, pupae) as a fixed factor. Colony of origin and colony number were used as random factors (Weekly brood model: count ~ treatment*brood stage*week + (1|original colony) + (1|colony number)). The proportional brood distribution between treatment was assessed for each brood stage using a test of Equal or Given Proportions. For all brood analysis, the first week was omitted due to the remaining influence of the stock colonies on the queen as well as the expected absence of effect from the feeding treatment. Moreover, five experimental nests (nest 7: CO; nest 24: GL; nest 38 and 47: HT; nest 66: HTGL) were omitted analysis due to the death of the two queens.

The abundance of ROS in relation to protein content was assessed with a Kruskal–Wallis test. The quotient from the measured fluorescence and absorbance were used as the response variable and the treatments as fixed effect. Seven samples had extreme values and were tested as outliers by the Univariate Outliers Using Boxplot Methods from the *rstatix* package (Kassambara 2021) and therefore were excluded from the analysis (nest 8: CO; nests 19, 21 and 22: GL; nest 28: LT; nest 37: HT; nest 53: LTGL).

The density of *Wolbachia* was assessed using general linear mixed models from the *glmmTMB* package and a Kruskal–Wallis test for *Westeberhardia*. Threshold cycle (Ct) values were normalized to the reference gene EF1. The ∆Ct-values were transformed to log(2 − ∆Ct) for clarity. For *Wolbachia*, a gaussian distribution was used with the log values as response variable, with feeding treatment as a fixed factor. Colony of origin and colony number were used as random factors (CoxA abundance: log(2 − ∆Ct) ~ treatment + (1|original colony) + (1|colony number)). For *Westeberhardia*, no random effect could be considered with the statistical test. To compare the treatments, a pairwise comparisons with Wilcoxon rank-sum test was conducted with a Bonferroni´s p-value adjustment. Nest 46 (treatment: HT) was not sampled due to insufficient number of workers.

The TiO_2_ concentration in the ant body was analysed with a linear mixed model with a Gaussian distribution. Concentration was used as the response variable, feeding treatment as the fixed factor and colony of origin and colony number were used as random factors (TiO_2_ concentration: concentration ~ treatment + (1|colony number/original colony)). Titanium dioxide was only detected in HT and HTGL and therefore these only two treatments were included in the statistical analysis. Moreover, three samples were removed from the analysis as they were tested as outliers (sample 1 and 4: HT; sample 8: HTGL) using the interquartile range.

The morphometric data were analysed with linear mixed models, one model per trait, using Gaussian distribution. Measurement was used as the response variable, feeding treatment as the fixed factor and the treatment colony was used as random factor (*Trait* measurement: *trait size* ~ treatment + (1|treatment_colony)). The two head measurements did not follow a normal distribution; therefore, they were analysed with a Kruskal–Wallis test. In that case, the treatments were compared with a pairwise comparisons with Wilcoxon rank-sum and a Bonferroni´s p-value adjustment.

## Results

### Worker and queen survival and brood count

No differences in mortality were observed among the six different diets (X^2^ = 3.200, df = 5, *p* = 0.669; suppl. config, 1). Similarly, the queen survival showed not significantly association with the dietary treatments (X^2^ = 3.084, df = 5, *p* = 0.687; suppl. config. 2).

**Fig. 4:**
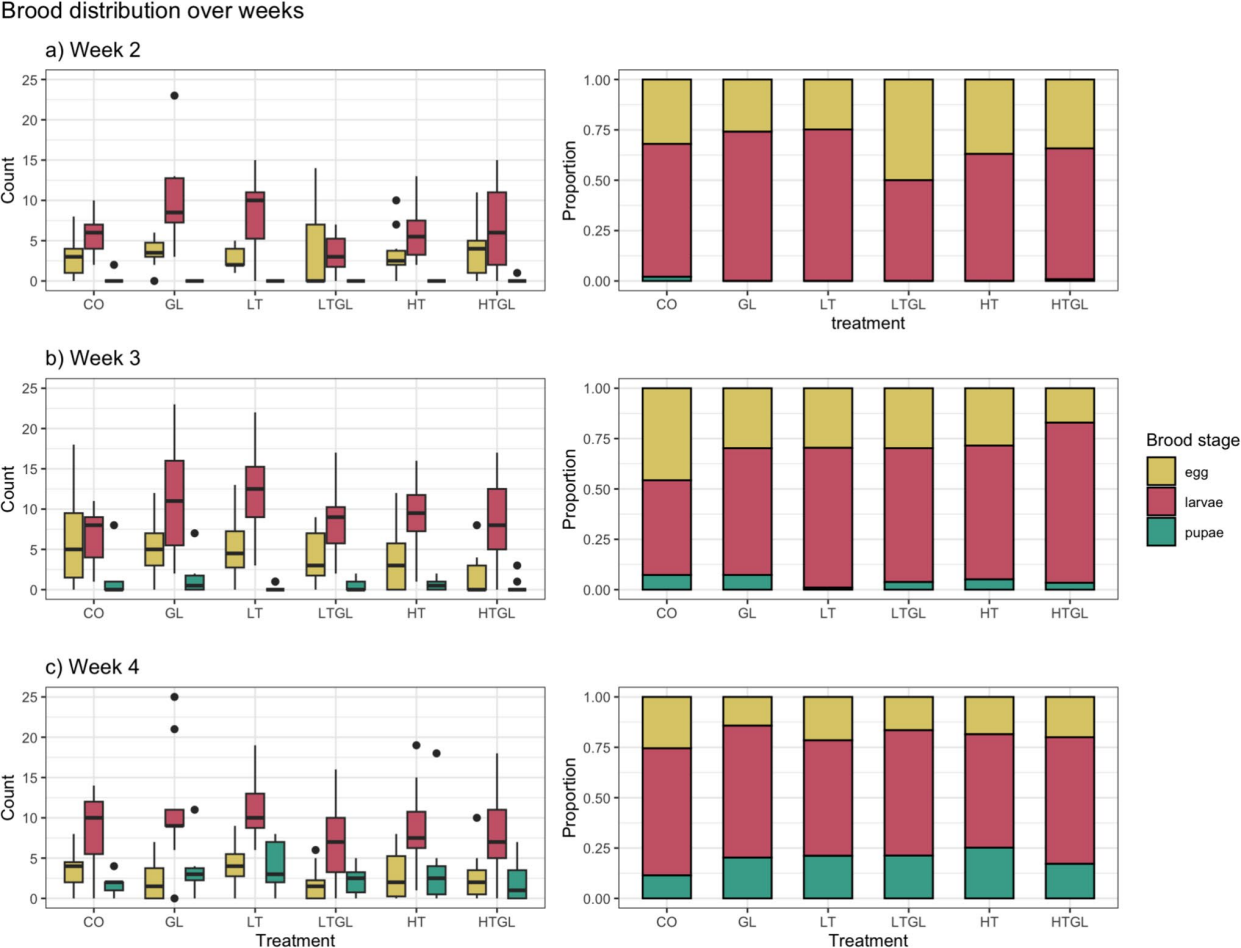
Brood count and proportion on week **a** 2, **b** 3 and **c** 4. The error bars represent the 05% CI. CO: Control; GL: Glyphosate; HT: High TiO_2_NPs; HTGL: High TiO_2_NPs with glyphosate; LT: Low TiO_2_NPs; LTGL: Low TiO_2_NPs with glyphosate. N_CO and HTGL_ = 11; N_GL and HT_ = 10; N_LT and LTGL_ = 12

The brood production did not significantly differ between treatments (X^2^ = 18.826, df = 13, *p* = 0.1286) but it did between brood stages (X^2^ = 368.075, df = 10, *p* < 0.0001) and weeks (X^2^ = 54.235, df = 6, *p* < 0.0001). There was significantly less eggs than larvae (*p* < 0.0001) and more total brood in week 4 than week 3 (*p* = 0.0002). The brood count of week 1 is displayed in supplementary configure 3 where the number of eggs was higher than larvae. There was no interaction between treatments and brood stages (X^2^ = 14.884, df = 14, *p* = 0.3862) or weeks (X^2^ = 17.590, df = 14, *p* = 0.2261) nor between treatments, brood stages and weeks (X^2^ = 16.136, df = 20, *p* = 0.7081). There was a significant interaction between brood stages and weeks (X^2^ = 60.581, df = 8, *p* < 0.0001). Contrary, to absolute count, proportional brood stage distribution was altered by the treatments (Fig. 4, right) compared to the control group. On week 3, most treatments have different proportion of eggs and larvae and on week 4, this difference was only observable for pupae where the proportion of pupae is significantly smaller in CO than any other treatment except HTGL. Results are exposed in supplementary Table 3.

### Reactive oxygen species content and endosymbiont densities

There was no significant effect of the feeding treatments on the H_2_O_2_ (X^2^ = 2.8082, df = 5, *p* = 0.7295) in ants.

**Fig. 5:**
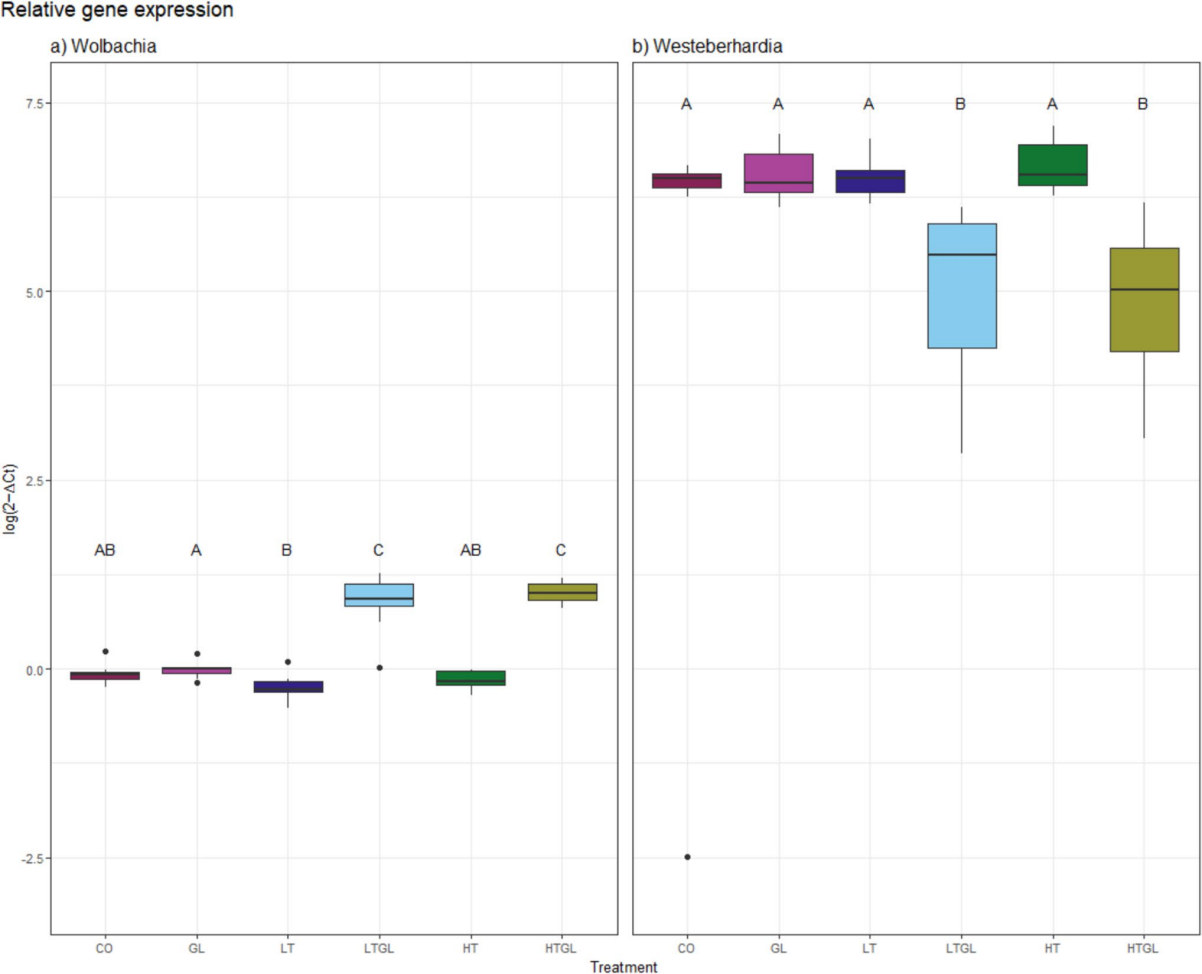
Relative densities of the endosymbionts **a** Wolbachia and **b** Cand. Westeberhardia in workers. The error bars represent the 05% CI. Pairwise comparisons indicated with letter. CO: Control; GL: Glyphosate; HT: High TiO_2_NPs; HTGL: High TiO_2_NPs with glyphosate; LT: Low TiO_2_NPs; LTGL: Low TiO_2_NPs with glyphosate. N_treatment_ = 12, except for GL and HT: *N* = 11

There was a significant difference in bacteria density due to the feeding treatment (Fig. 5, *Wolbachia:* X^2^ = 634.360, df = 5, *p* < 0.0001; *Westeberhardia*: X^2^ = 43.49, df = 5, *p* < 0.0001) in the ants. The pairwise comparison tests showed that the density increased for *Wolbachia* and decreased for *Westeberhardia* when TiO_2_ is combined with glyphosate, regardless of the TiO_2_ concentration (p´s < 0.003). Interestingly, there was also a significant difference between GL and LT for *Wolbachia* (*p* = 0.037).

### Distribution of elements in the ant body

**Fig. 6:**
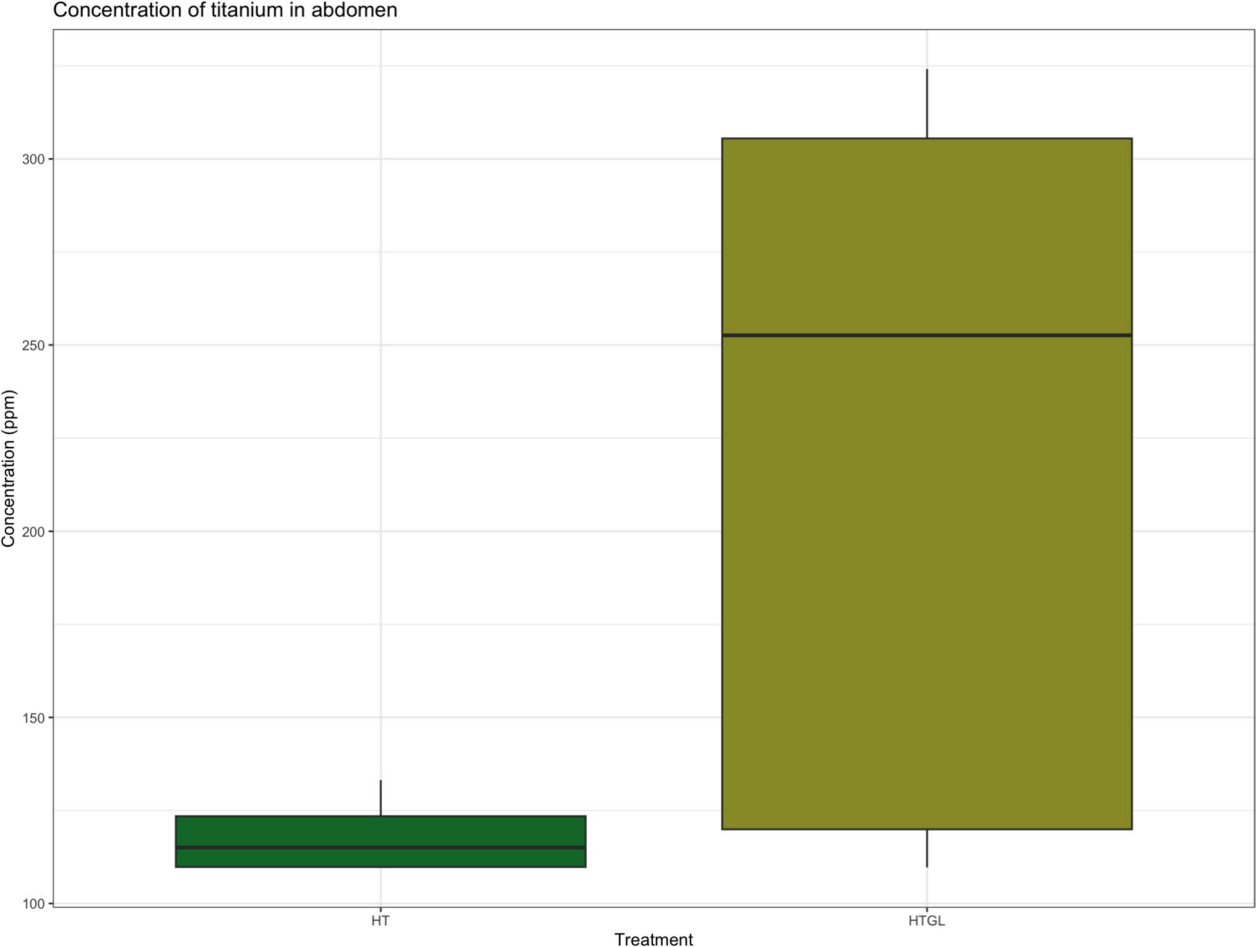
Concentration of titanium in abdomens. The error bars represent the 05% CI. HT: High TiO2NPs; HTGL: High TiO_2_NPs with glyphosate. N_HT_ = 4 and N_HT_ = 5

No significant titanium signals were detected in CO, GL, LT, or LTGL groups. In contrast, Ti was predominantly localised in the abdominal region of ants from the HT and HTGL groups. Results suggest an increased accumulation of Ti in the HTGL group (X^2^ = 5.1976, df = 1, *p* = 0.022), where glyphosate was combined with TiO_2_NPs (Fig. 6).

### Morphometric measurements

**Fig. 7:**
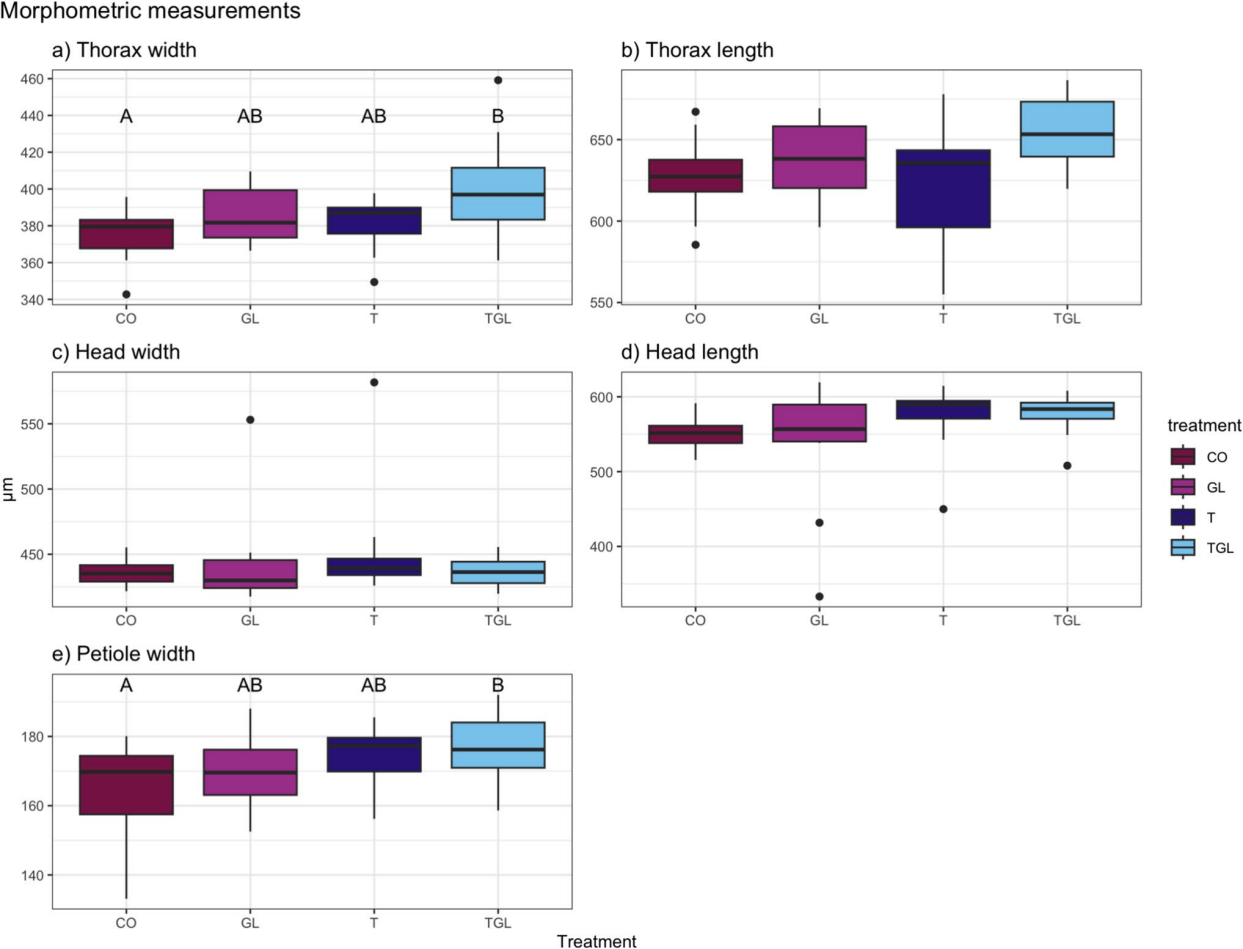
Morphometric traits of virgin queen produced in colonies subjected to feeding treatments. **a**) Thorax width, **b**) Thorax length, **c**) Head width, **d**) Head length and **e**) Petiole width. The error bars represent the 05% CI. Pairwise comparisons are indicated with letter. CO: Control; GL: Glyphosate; T: TiO_2_NPs; TGL: TiO_2_NPs with glyphosate. N_treatment_ = 12, except for GL: *N* = 16

The combination of glyphosate and TiO_2_ had an effect on the morphology of newly produced virgin queens compared to the control treatment (Fig. 7). The thorax width and length (TW and TL), the petiole width (PW) and the head length (HL) were significantly altered by the feeding treatments (Table 1). The pairwise tests showed an increase in size of the TW and PW when fed with TGL compared to CO, it was almost significant for the TL as well (TW: *p* = 0.055; TL: *p* = 0.078; PW: *p* = 0.037). By doing a pairwise comparison between treatments and CO, we did get a significant p-value for TL (*p* = 0.047). The HL was significantly affected by the treatments, yet the pairwise comparison did not show any significant results. Nevertheless, the p-value for T-CO is 0.06, indicating a possible effect of TiO_2_NPs.

**Table 1:** Effect of diet treatments on virgin queen specific trait measurement

	Chisq	Df	Pr (> Chisq)
Thorax width	7.811	3	0.050
Thorax length	10.844	3	0.017
Petiole width	9.268	3	0.026
Head width	2.295	3	0.513
Head length	8.256	3	0.041

## Discussion

In the current study, we demonstrate sublethal effects of a four-week chronic exposure to glyphosate and TiO_2_NPs in the ant *C. obscurior* using field-realistic concentrations. The two stressors alone or in combination were non-lethal nor increased the production of ROS. However, other sublethal effects were observed such as an alteration of the proportional brood distribution with more pupae towards the end of the observation period in the presence of a stressor. Moreover, a synergistic effect is observed between TiO_2_NPs and glyphosate regarding gut symbiont densities, newly produced queen size and tendency of the bioaccumulation of titanium in the gut. The findings emphasize the significance of examining various stressors and prolonged exposure, as subtle long-term effects that may not be immediately apparent could still have significant impacts on overall fitness.

The used concentration of glyphosate, TiO_2_NPs or their combination did not increase mortality of the ants in this experiment. Indeed, glyphosate targets specifically an enzyme that is not present in insects preventing the molecule to disrupt critical biochemical processes within them (Cole 1985). Nevertheless, multiple studies have shown increase in mortality when exposed to glyphosate (see review form Battisti et al. 2021). Leponiemi et al. (2022) reported no effect of glyphosate on survival in *C. obscurior* over a period of 14 days in concentrations ranging from 0 to 1000µg/g. Glyphosate appears not to have acute toxicity on *C. obscurior* while chronic exposure leads to subtle but significant long-term effects. TiO_2_NPs is expected to be toxic to insects as NPs are known to produce antioxidant and detoxifying enzymes, act as trypsin inhibitors, decrease membrane permeability and bind to the insect cuticle (see review from Benelli 2018). In TiO_2_NPs, increased mortality was observed in *G. pyloalis* (Lepidoptera; Memarizadeh et al. 2014) but no increase was observed in *O. fasciatus* (Hemiptera), *B. mori* (Lepidoptera), or *D. melanogaster* (Diptera; López-Muñoz et al. 2019; Xie et al. 2014; Jovanović et al. 2016 respectively). Dissimilarities in the responses could stem from variations in the nanoparticle properties (e.g., size, surface, etc.) and concentration, as well as on the specific sensitivity of the insects, the method of administration, or the developmental stage utilized in each experiment.

ROS is often produced following exposure to pollutants (Nathan and Cunningham-Bussel 2013) including glyphosate (de Aguiar et al. 2016; Feng et al. 2022; Pal et al. 2022) as well as TiO_2_NPs (Federici et al. 2007; Tuncsoy and Mese 2021). Excessive ROS production can cause oxidative stress, which damages cellular component, and impair various physiological functions including development and reproduction (Tonogawa et al. 2021). However, in *O. fasciatus* ingestion of TiO_2_NPs did not increase oxidative stress either (López-Muñoz et al. 2019). In this study, we measured oxidative stress as the production of H_2_O_2_ in relation to protein content. The fact that glyphosate and TiO_2_NPs did not induce oxidative stress may be because the concentration of stressors in the diet is not sufficient to induce the production of H_2_O_2_.

Leponiemi et al. (2022) showed that glyphosate potentially lowers reproductive performance of *C. obscurior* queen when exposed to 100μg/g, about 20 times higher concentration as used in this experiment. Moreover, the feeding treatments lasted 12 weeks, 3 times longer. We did not observe a change in brood production. Therefore, the effect is potentially linked to the concentration of the glyphosate as well as exposure time. We did observe an effect on the proportional brood distribution by the end of the observation period, indicating a possible interaction with exposure time. Nevertheless, the experimental set-up does not allow for a straightforward reasoning. A possible explanation is an increase in developmental time as observed in other insects when ingesting TiO_2_NPs (Zorlu et al. 2018; López-Muñoz et al. 2019), glyphosate (Schneider et al. 2009; Vazquez et al., 2018) or combined stressors (Vommaro et al. 2024).

In our experiment, newly produced queens had bigger thorax and petiole when the stressors were combined compared to the control group. The size of ant queen's thorax is often assumed to be directly associated with the mass of her flight muscles, given that the flight muscles occupy most of the thorax (Keller et al. 2014). The size of these flight muscles may reflect the ant queen's dispersal ability. Following dispersal, the flight muscles are typically utilized for resources during the initial stages of nest founding (Peeters and Fuminori 2001). It can be speculated that exposure to the combination of glyphosate and TiO_2_NPs may indicate unfavourable conditions, prompting a shift towards investment in dispersal or colony-founding ability, potentially similar to the production of winged males in *C. obscurior *colonies under stressful conditions (Cremer and Heinze 2003). The increase in thorax size and the associated developmental changes come with higher energetic costs. This could impact the overall productivity and health of the colony, especially under prolonged stress conditions Bordoni et al. 2017). Interestingly, the thorax was larger in *C. obscurior* queen with glyphosate alone (Leponiemi et al. 2022) at higher concentration, therefore, it can be hypothesized that the concentration used for this experiment of glyphosate or titanium alone was not inducing a big enough stress response that was observed in combination.

Both stressors have been shown to alter the gut microbes in insects (Li et al. 2020; Papa et al. 2021 for titanium and Motta et al. 2018; Blot et al. 2019 for glyphosate). Alterations in the gut microbiota of insects can have profound effects on their nutrition, development and behavior, which in return influence their survival, reproduction, and interactions with pathogens (Engel and Moran 2013). However, this was not the case in our results where alone, the stressors did not affect the gut microbes. Nevertheless, it is consistent with Leponiemi et al. (2022) where glyphosate did not alter endosymbiont in *C. obscurior*. When combined, regardless of the titanium concentration, the density of the symbionts was altered. The differential effects on the density of *Wolbachia* (increase) and *Cand. Westeberhardia* (decrease) within the ant gut could be explained by factors such as competition dynamics within the microbiome, differential sensitivity to stressors, and distinct ecological roles or metabolic demands of these bacteria. For instance, *Cand. Westeberhardia* could be more sensitive to the stressors, lowering competition pressure on *Wolbachia* creating gut dysbiosis (Rosenfeld et al., 2017). *Wolbachia* is a maternally inherited endosymbiotic bacteria that is often considered to be a manipulator of reproductive strategies (Ün et al. 2021), an increase of its density could impact evolutionary dynamics of the colony and be potentially linked with the production of bigger queens. *Cand*. *Westeberhardia* is known to contribute to cuticle formation (Klein et al. 2016). Therefore, the observed lowered density could potentially increase the ant vulnerability to stresses due to improper cuticle formation. All in all, the bacteria densities alteration observed in this research requires further investigation to unravel the direct effect on the ant fitness.

Our results strongly suggest a synergism between TiO_2_NPs and glyphosate. Synergism has been reported between TiO_2_NPs and diverse insecticides such as cypermethrin in rats (Li et al. 2023) and recently in honeybees with deltamethrin (Shahzad and Manzoor 2024). A possible explanation for this synergism is due to the interaction of NPs with the pesticide as TiO_2_NPs is known to adsorb glyphosate (Marconi et al. 2025) and may enhance its effects. It has been shown in aqueous environments that the phosphonate component of the pesticide facilitated the attachment of glyphosate to the NP, while the carboxylic component contributed to the electrostatic stabilization of the NP. Additionally, TiO_2_NPs were found to enhance the breakdown of glyphosate into AMPA and to attach to it (Ilina et al. 2017). AMPA is a type of antagonist for the glutamate receptor that mediates fast synaptic transmission (Bleakman and Lodge 1998). Using an innovative approach such as LA-ICP-TOFMS allowed us to observe and calibrate a possible accumulation of titanium in the gut of our ants when combined with glyphosate. This bioaccumulation in the gut might have contributed to the observed physiological and morphometric changes (Ding et al. 2020; Zhang et al. 2023) and possible further parameters could have been impacted outside the scope of this research such as locomotor behavior (Zhang et al. 2023) or immune system impairment (Mir et al. 2020). Moreover, it would be interesting to quantify glyphosate in the ant´s body in the future and to investigate the effect of glyphosate-based herbicides as their toxicity varies considerably (Roddam et al. 2025).

Increased reports on insect losses and the accelerating use of nanomaterials call for more studies to investigate synergistic effects. In this experiment, we report the findings of short term, yet chronic oral exposure to TiO_2_NPs and glyphosate, alone and in combination. We focussed on both lethal and sublethal effects. We showed that in combination they have stronger effects on *C. obscurior* than individually, this is the first reported account of synergism between TiO_2_NPs and glyphosate in ants. The measured parameters are directly related to individual and overall colony fitness. Further investigations are required to understand their direct consequences on colony development and survival in a natural context. We clearly demonstrate that field-realistic doses of these agricultural pollutants lead to several sublethal effects in our study organism which could reduce fitness and indicate reasons behind the disappearance of insect in environment. In addition to distinct physiological effects, soil pollution affects habitat selection of ground-dwelling insects (Segev et al. 2023) and simplify food webs (Morales-Silva et al. 2023) leading to a reduction of biodiversity and an alteration of the ecological dynamics of insect populations. It is clear that more long-term studies under controlled exposure to various pollutants is needed to understand the health risks such molecules exhibit in insects and how it threatens biodiversity.

## Supplementary Information

Below is the link to the electronic supplementary material.

ESM 1 (DOCX 210 KB)

## Data Availability

Data will be made available on request.
