# Supplementary material for: Fitness related effects of titanium dioxide nanoparticles and glyphosate exposure on Cardiocondyla obscurior
Source: Environ Sci Pollut Res Int. 2026 Jan 23;33(5):1622–38. doi: 10.1007/s11356-025-37388-y (PMC12901099; doi:10.1007/s11356-025-37388-y)
Supplement: Supplementary file 1 — (DOCX 210 KB) [file 11356_2025_37388_MOESM1_ESM.docx]

# Supplementary information - Fitness related effects of titanium dioxide nanoparticles and glyphosate exposure on Cardiocondyla obscurior

Danae Nyckees*^1^, Raquel Gonzalez de Vega^2^, Reinhard Sittinger^2^, David Clases^2^ and Dalial Freitak^1^

^1^Institute of Biology, Karl-Franzens-Universitat Graz, Graz, Austria

^2^Institute of Chemistry, Karl-Franzens-Universitat Graz, Graz, Austria

*Corresponding author: danae.nyckees@uni-graz.at / +43 (0)316 380 – 3915

## Supplementary material

**Supplementary table 1** Testing the effect of ant replacement after one week of acclimation

| Model: lm(replaced ants ~ treatment) | | | | |
| --- | --- | --- | --- | --- |
| Anova Table (Type II tests) | | | | |
| Response: replaced ants | | | | |
|  | Sum Sq | Df | F value | Pr(>F) |
| treatment | 41.24 | 5 | 0.8326 | 0.5312 |
| Residuals | 653.75 | 66 |  |  |

**Supplementary table 2** Primers

| Target organism | Target gene | Primer | Sequence | Reference |
| --- | --- | --- | --- | --- |
| Wolbachia | *cytochrome* | coxA_forward | TTGGTCATCCAGAAGTTTACGT | Ün *et al.* 2021 |
|  | *oxidase* | coxA_reverse | TGAGCCCAAACCATAAAGCC |  |
| *Cand.* | *nrdb* | nrdB_forward | GGAAGGAGTCCTAATGTTGCG | Klein *et al.* 2016 |
| Westeberhardia |  | nrdB_reverse | ACCAGAAATATCTTTTGCACGTT |  |
| *C.obscurior* | *elongation factor* | EF1_forward | TCACTGGTACCTCGCAAGCCGA | Klein *et al.* 2016 |
| (reference) | *1-alpha 1* | EF1_reverse | AGCGTGCTCACGAGTTTGTCCG |  |

**Supplementary table 3** Proportional brood distribution compare to the control group.

| Week | Treatment | proportions1 | proportions2 | pval | CImin | CImax | Brood stage |
| --- | --- | --- | --- | --- | --- | --- | --- |
| 2 | GL | 0.319588 | 0.259259 | 0.391397 | -0.06717 | 0.18783 | egg |
| 2 | GL | 0.659794 | 0.740741 | 0.234111 | -0.20961 | 0.04772 | larvae |
| 2 | GL | 0.020619 | 0 | 0.339211 | -0.01652 | 0.057756 | pupae |
| 2 | LT | 0.319588 | 0.248062 | 0.299694 | -0.05653 | 0.199578 | egg |
| 2 | LT | 0.659794 | 0.751938 | 0.171854 | -0.22136 | 0.03707 | larvae |
| 2 | LT | 0.020619 | 0 | 0.357224 | -0.01669 | 0.057928 | pupae |
| 2 | HT | 0.319588 | 0.369565 | 0.568825 | -0.19599 | 0.096037 | egg |
| 2 | HT | 0.659794 | 0.630435 | 0.787454 | -0.11768 | 0.176396 | larvae |
| 2 | HT | 0.020619 | 0 | 0.500629 | -0.01825 | 0.059487 | pupae |
| 2 | LTGL | 0.319588 | 0.5 | 0.021469 | -0.33422 | -0.0266 | egg |
| 2 | LTGL | 0.659794 | 0.5 | 0.044352 | 0.00501 | 0.314577 | larvae |
| 2 | LTGL | 0.020619 | 0 | 0.55251 | -0.01891 | 0.06015 | pupae |
| 2 | HTGL | 0.319588 | 0.342105 | 0.841853 | -0.15932 | 0.114286 | egg |
| 2 | HTGL | 0.659794 | 0.649123 | 0.986274 | -0.12757 | 0.148914 | larvae |
| 2 | HTGL | 0.020619 | 0.008772 | 0.887862 | -0.03075 | 0.054443 | pupae |
| 3 | GL | 0.456954 | 0.297753 | 0.004176 | 0.049035 | 0.269366 | egg |
| 3 | GL | 0.470199 | 0.629213 | 0.00541 | -0.27178 | -0.04625 | larvae |
| 3 | GL | 0.072848 | 0.073034 | 1 | -0.05676 | 0.056385 | pupae |
| 3 | LT | 0.456954 | 0.296117 | 0.002624 | 0.054105 | 0.267569 | egg |
| 3 | LT | 0.470199 | 0.694175 | 3.22E-05 | -0.33119 | -0.11677 | larvae |
| 3 | LT | 0.072848 | 0.009709 | 0.004232 | 0.01384 | 0.112438 | pupae |
| 3 | HT | 0.456954 | 0.284672 | 0.003802 | 0.055673 | 0.288891 | egg |
| 3 | HT | 0.470199 | 0.664234 | 0.001393 | -0.31321 | -0.07486 | larvae |
| 3 | HT | 0.072848 | 0.051095 | 0.604524 | -0.04069 | 0.084191 | pupae |
| 3 | LTGL | 0.456954 | 0.297468 | 0.005494 | 0.046267 | 0.272703 | egg |
| 3 | LTGL | 0.470199 | 0.664557 | 0.000855 | -0.30927 | -0.07945 | larvae |
| 3 | LTGL | 0.072848 | 0.037975 | 0.273802 | -0.02266 | 0.092402 | pupae |
| 3 | HTGL | 0.456954 | 0.17094 | 1.59E-06 | 0.17371 | 0.398317 | egg |
| 3 | HTGL | 0.470199 | 0.794872 | 1.27E-07 | -0.44038 | -0.20896 | larvae |
| 3 | HTGL | 0.072848 | 0.034188 | 0.27237 | -0.02186 | 0.099182 | pupae |
| 4 | GL | 0.255034 | 0.142857 | 0.017727 | 0.018105 | 0.206248 | egg |
| 4 | GL | 0.630872 | 0.654762 | 0.744612 | -0.13592 | 0.088143 | larvae |
| 4 | GL | 0.114094 | 0.202381 | 0.047467 | -0.17397 | -0.0026 | pupae |
| 4 | LT | 0.255034 | 0.215517 | 0.44216 | -0.05373 | 0.132764 | egg |
| 4 | LT | 0.630872 | 0.573276 | 0.312023 | -0.04819 | 0.163379 | larvae |
| 4 | LT | 0.114094 | 0.211207 | 0.021135 | -0.17587 | -0.01836 | pupae |
| 4 | HT | 0.255034 | 0.18543 | 0.188269 | -0.03056 | 0.169763 | egg |
| 4 | HT | 0.630872 | 0.562914 | 0.279256 | -0.04945 | 0.185365 | larvae |
| 4 | HT | 0.114094 | 0.251656 | 0.003395 | -0.23023 | -0.04489 | pupae |
| 4 | LTGL | 0.255034 | 0.165354 | 0.096115 | -0.01286 | 0.192223 | egg |
| 4 | LTGL | 0.630872 | 0.622047 | 0.979068 | -0.11299 | 0.13064 | larvae |
| 4 | LTGL | 0.114094 | 0.212598 | 0.039099 | -0.19337 | -0.00364 | pupae |
| 4 | HTGL | 0.255034 | 0.2 | 0.330251 | -0.04827 | 0.158338 | egg |
| 4 | HTGL | 0.630872 | 0.628571 | 1 | -0.1114 | 0.116002 | larvae |
| 4 | HTGL | 0.114094 | 0.171429 | 0.219675 | -0.14491 | 0.030236 | pupae |


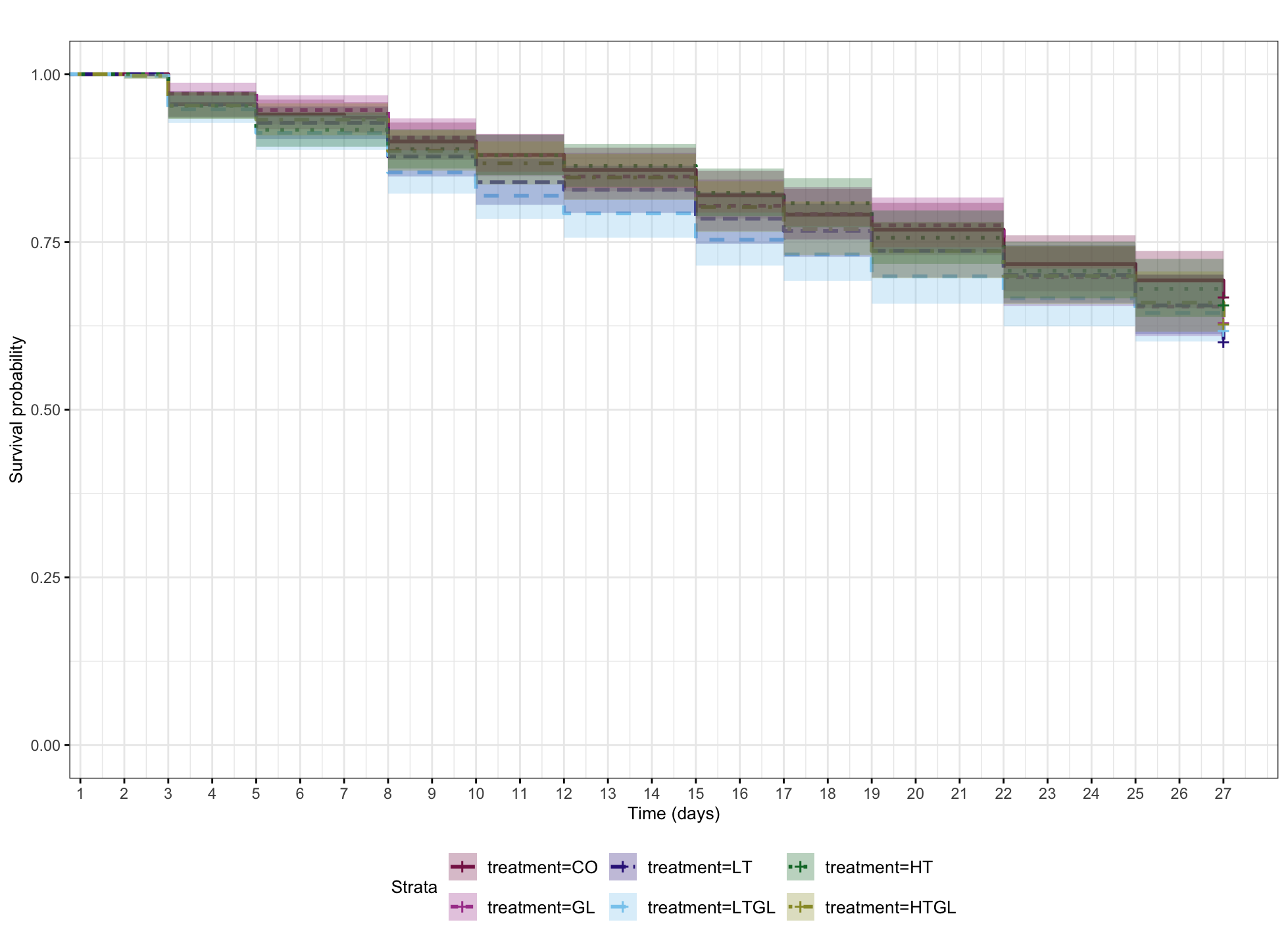


**Supplementary fig. 1** Daily survival probability. The shaded areas represent the 05% CI. CO: Control; GL: Glyphosate; HT: High TiO_2_NPs; HTGL: High TiO_2_NPs with glyphosate; LT: Low TiO_2_NPs; LTGL: Low TiO_2_NPs with glyphosate. N_treatment_ = 12, except for GL: N = 11


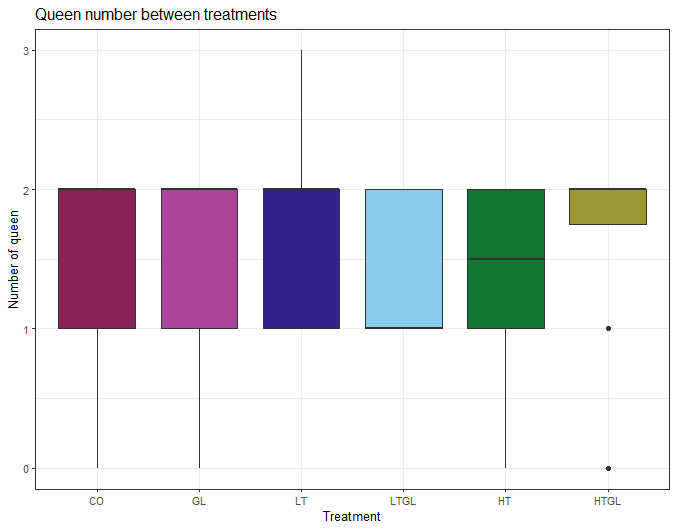


**Supplementary figure 2** Queen number at the end of the 4 weeks. CO: Control; GL: Glyphosate; HT: High TiO_2_NPs; HTGL: High TiO_2_NPs with glyphosate; LT: Low TiO_2_NPs; LTGL: Low TiO_2_NPs with glyphosate. N_treatment_ = 12, except for GL: N = 11


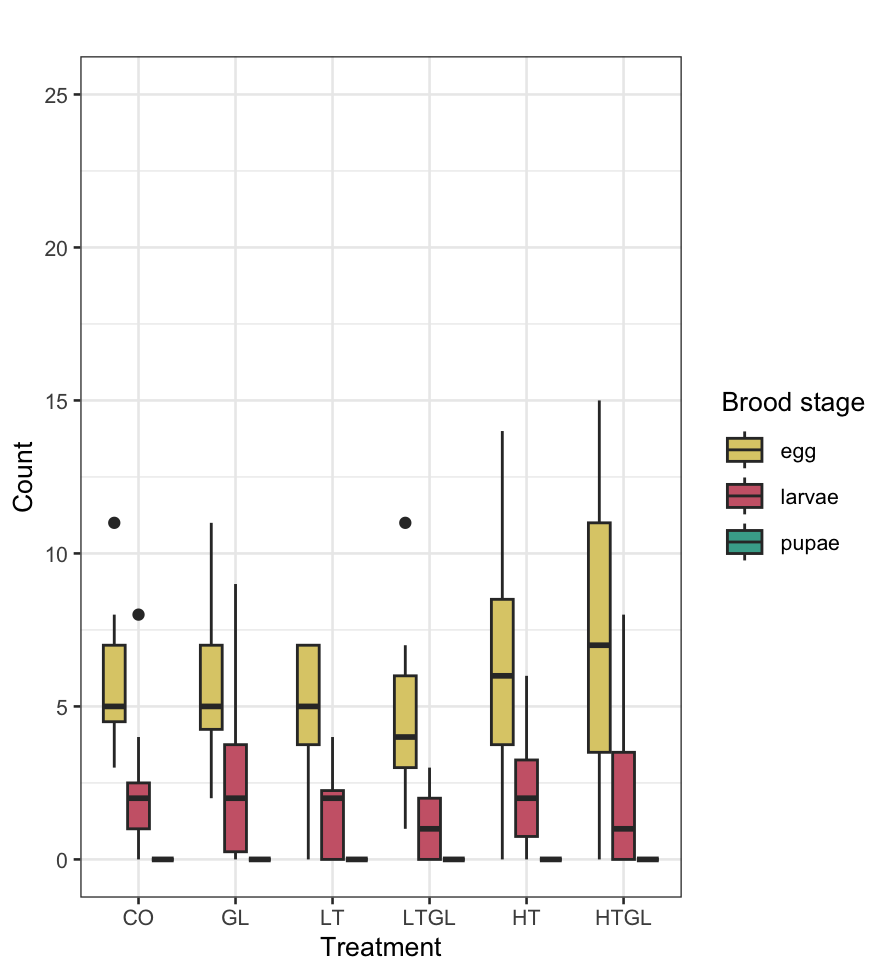


**Supplementary fig. 3** Brood count per stage at week 1. The error bars represent the 05% CI. CO: Control; GL: Glyphosate; HT: High TiO_2_NPs; HTGL: High TiO_2_NPs with glyphosate; LT: Low TiO_2_NPs; LTGL: Low TiO_2_NPs with glyphosate. N_CO and HTGL_ = 11; N_GL and HT_ = 10; N_LT and LTGL_ = 12
